# Exploring Policy Change in the Emergency Department: A Qualitative Approach to Understanding Local Policy Creation and the Barriers to Implementing Change

**DOI:** 10.7759/cureus.3086

**Published:** 2018-08-02

**Authors:** Sameer Shaikh, Tara Stratton, Alim Pardhan, Teresa M Chan

**Affiliations:** 1 Anesthesia, Trillium Health Partners, Ontario, CAN; 2 Emergency Medicine Training Program, McMaster University, Ontario, CAN; 3 Emergency Medicine, McMaster University, Ontario, CAN; 4 Faculty of Health Sciences, McMaster University, Hamilton, CAN

**Keywords:** policy development, knowledge translation, implementation science, qualitative methods

## Abstract

Introduction

With thousands of new medical trials released every year, health care policymakers must work diligently to incorporate new evidence into clinical practice.

Although there are some broad conceptual frameworks for knowledge translation in the emergency department (ED), there are few user-centered studies that illustrate how local policymakers develop and disseminate new policies.

Objectives

Our study sought to evaluate the process by which new departmental policies are formed in ED, how new evidence was integrated into this process, and to explore barriers to implementation.

Methods

Semi-structured interviews were conducted with local administrators from nine major hospitals in Ontario, Canada. Interviews were transcribed and qualitative data was analyzed using constructivist grounded theory.

Results

Five broad steps in the policy creation process were identified: 1) Problem identification and motivation for change; 2) building a policy team; 3) policy construction; 4) implementation and monitoring of new departmental policies; 5) actively addressing barriers to the ED policymaking process. Common sub-themes in each of these categories were highlighted. Four main themes also emerged regarding barriers experienced in policymaking: Education and knowledge transfer; lack of a change culture; resource limitations; and cumbersome bureaucratic structures.

Conclusion

Our study identified common facilitators and barriers that policymakers face in their ability to create health policy in the ED. While local context influences the policymaking process, a standardized framework would ensure a more systematic approach for policymakers and allow scientists to better understand how evidence is integrated at the local level.

## Introduction

Health practitioners and policymakers are frequently faced with the challenge of integrating expansive amounts of new medical literature into their practice. In 2010, Bastian et al. reported that nearly 75 trials and 11 systematic reviews of trials were being published on a daily basis [1]. Undoubtedly, that number will continue to grow throughout the 21st century as billions of dollars worldwide go to medical research programs [2]. Although keeping up with newly released medical research is difficult, it is further compounded by the equally, if not more, difficult task of changing local healthcare practices to match the latest guidelines.

To ensure that patients receive the highest quality of care, emergency departments (EDs) utilize a wide array of policies and guidelines that influence healthcare decisions and ensure consistency [3]. While there are some conceptual frameworks for translating new knowledge into the clinical environment, many of these frameworks usually cater to high-level policy generation in the public health sphere or at the level of regional or national governments. At a hospital-specific level, there is also no consistent gold-standard approach that centers apply [4,5]. Most clinicians in the ED are focused on providing evidence-based, best-practice care to their patients, but when it comes to health policymakers the exact process of policy creation is often unclear. To date, few qualitative studies have been conducted to better understand how local policymakers develop and disseminate policies that are responsive to new best-practice clinical evidence or to the evolving demands and capabilities of local hospitals.

The problem

Although the concept of continually evolving to match current literature is ideal, there are many barriers to this process. These barriers can be broken into three broad categories: knowledge, attitudes, and internal/external barriers [6]. A lack of awareness or familiarity of the new medical knowledge in question often forms the basis of knowledge barriers, whether from lack of engagement with reading new literature, a lack of scientific literacy, or a lack of critical appraisal capabilities. In terms of attitudes as a barrier, this may manifest as a disagreement with the method, content or applicability of literature from personal biases, a lack of expectant gains through the proposed changes, or perceived disadvantages of the proposed change on the individual’s practice [6]. Internal barriers may be related to lack of established pathways or support to enact policy change and factors associated with knowledge use as an innovation (e.g., perception that innovation is different from a clinician’s personal approach), whereas external barriers include environmental factors (e.g., insufficient time, materials, administrative support) and patient factors (e.g., patient preferences) [6].

Incorporating end-user perspectives into conceptual frameworks for knowledge translation may assist in the change management process [7]. Simply put, understanding local practices of smaller institutions and how they derive local policies may provide insights for what issues scientists should bear in mind when attempting to guide others on how to integrate their new knowledge into practice.

Although there are a number of theoretical models that identify the process of transforming new knowledge into policy, it is unclear what models (if any) local emergency departments utilize in practice. Furthermore, there is a scarcity of literature on specific barriers to implementing change in emergency departments in Canada.

Our study looked to qualitatively explore the environment of departmental policy creation in EDs in our Local Health Integrated Network (LHIN). The objective of our study was twofold: 1) To evaluate the process by which new departmental policies are formed in the ED; 2) To explore barriers and possible solutions to implementing new emergency department policies.

## Materials and methods

Study design

This was a qualitative study that involved interviews with local administrators and clinicians who participated in ED departmental policy creation at their institution. REB approval was granted from the Hamilton Integrated Research Ethics Board and approval to conduct this research was given from the local Emergency Services Steering Committee (ESSC) in the Hamilton-Niagara-Haldimand-Brant (HNHB) Local Health Integration Network (LHIN). We abided by the Consolidated criteria for reporting qualitative research (COREQ) guidelines [8].

Funding

This study was funded via a grant from the HNHB LHIN.

Study setting and population

The study was conducted in the HNHB LHIN. The LHIN is a regional governance body that connects nine hospital systems in our region within Ontario, Canada. The nine EDs are: Brantford General Hospital, Joseph Brant Hospital, Haldimand War Memorial Hospital, West Haldimand General Hospital, St. Joseph’s Healthcare Hamilton, Hamilton Health Sciences, Norfolk General Hospital, and Niagara Health Systems. The research team consisted of one ER resident (SS), one medical student (TS), and two ER staff physicians (AP and TC).

Through the local ESSC, study investigators were introduced to administrators and clinicians involved with departmental policy creation in their local EDs. We attempted to intentionally sample across the various stakeholder groups. Participants received e-mailed invitations that described the goals of the study and asked them to participate in individual interviews. All participants provided verbal and written consent and voluntarily participated in this study. No participants withdrew from the study after providing consent. There were no repeat interviews.

A single, trained investigator (SS) conducted semi-structured interviews with participants from all the hospitals in our LHIN. The investigator did not hold any perceived position of power or influence over any of the participants and was a male resident physician. He had no previous qualitative research experience, but was trained via the senior author (TC) to conduct semi-structured interviews. Cross-checking was completed by the senior author to ensure the rigour of his interviews. Larger hospital systems had multiple study participants whereas smaller centers were represented by one participant.

The goal of the interviews was to conduct an in-depth exploration of how policymakers within our LHIN developed and disseminate new ED policies, as well as explore the various barriers they faced. Interviews were face-to-face 30 to 45 minutes in length and were conducted at times and locations convenient to the participants. All interviews were audio recorded and transcribed for subsequent analysis.

Data analysis

Research Team

Data analysis was primarily conducted by four members of the research team – one medical student (TS), one resident physician (SS), one attending physician with graduate-level qualitative research training and experience (TC) and one attending with graduate-level administrative training (AP). The analytic team was trained by the senior author (TC) to conduct the analyses of our data via a number of meetings, simulated interviews (to ensure that the interviewers could follow the ebbs and flows of a semi-structured interview), and guided analysis sessions.

Theoretical Approach

We utilized a constructivist grounded theory [9] approach to analyze the data. This qualitative technique guides us to iteratively examine our data primed with the knowledge from prior literature as we were hoping to build on the work that has been done in knowledge translation and implementation science, extracting themes/subthemes and then engaging in axial coding to generate a new conceptual framework about local policy development.

Transcript Management

Interviews were transcribed by a professional transcriptionist, and all transcripts were initially read without any notes. After all the transcripts were read, three researchers independently went through each transcript and identified key themes that related to our primary outcomes. Relevant themes were highlighted and the data were constantly compared, revised, and reduced as new data were examined.

Analysis

In consultation with the members of the research team, the resident and medical student researchers independently identified primary themes and developed memos regarding each topic area from the transcribed interviews. With the full investigatory team, these categories were compared, revised, expanded, and reduced until it was felt that saturation was achieved – i.e., when our team was unable to note any new themes. In an effort to achieve greater conceptual cohesion, we engaged in an axial coding activity to relate our newly uncovered categories and subcategories, attempting to reassemble our data in new ways after our initial open coding [9]. Finally, a narrative that encompassed all major themes and their subcategories was constructed. The final constructed analysis was emailed to our study participants for a member check to ensure that our final conceptual framework resonated with the participants.

## Results

A total of 15 semi-structured interviews were conducted with volunteer participants from all nine major hospitals in our local LHIN. Three of the participants were staff emergency physicians, whereas the remaining 12 participants were either administrators or other health care professionals that had an active role in departmental policy creation (i.e., nurses). Administrators varied in their clinical and administrative backgrounds with some having a predominantly clinical background and others having more financial expertise such as a Master of Business Administration (MBA). Table 1 displays the participant demographics.

**Table 1:** Participant demographics. F: Female; M: Male; MUMC: McMaster University Medical Center; HGH: Hamilton General Hospital; NHS: Niagara Health Systems; WLMH: West Lincoln Memorial Hospital.

Position	Number	Location
Emergency Physicians	4 (1 F, 3 M)	Joseph Brant, MUMC, HGH, Port Colborne
Registered Nurses	3 (All F)	West Haldimand, Haldimand War Memorial, Welland
Administrators	8 (All F)	NHS, WLMH, Joseph Brant, MUMC, Brantford, St. Catharine’s, St. Joseph’s Hamilton, HGH

**Figure 1:**
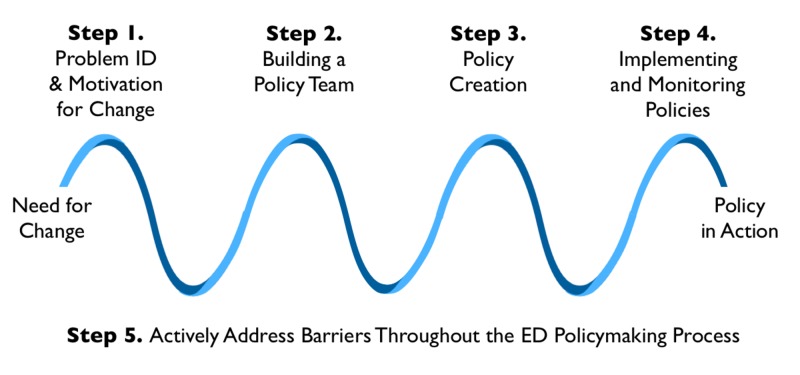
Five steps for emergency department (ED) policy development. A visual depiction of the five steps described by our participants for developing new emergency department policies.

Throughout our interviews, clinicians and administrators talked about a variety of themes required to develop new departmental policies. Although there were differences in approach to policy creation based on site, size, and experience, each concept expressed could be categorized into five major steps: 1) Problem identification and motivation for change, 2) building a policy team, 3) policy construction, 4) implementation and monitoring of new departmental policies, and 5) actively addressing barriers to the ED policymaking process. Figure 1 depicts the final conceptual framework developed by our analysis.

Table 2 displays some key exemplar quotes that highlight each of these steps.

**Table 2:** Five steps for Emergency Department Policy Development.

Step	Exemplar Quote
1. Problem Identification and Motivation for Change	“…[F]irst is the identification of the need. The need would be identified by many different stakeholders, front line providers in our emergency departments our partners [emergency medical services] for example, our police, they may also be a need identified by other internal stakeholders and I think for example our mental health populations. So there may be our mental health experts so they may be our mental health experts identify a policy opportunity for us here” - Participant 9 (Administrator)
2. Building a Policy Team	So from there, there would be a designated lead to develop a draft policy so what would happen is a working team would be brought together so it is usually a director, X may not do all of the policy, she may ask for a volunteer to take the lead on this specific policy so, massive transfusion protocols as an example where we've had maybe we could have had better co-ordination. So we've developed a massive transfusion protocol, we've had a working team together with a director as a lead, clinical educator, clinical manager, a couple of physicians, you know even spiritual health at the table. – Participant 13 (Nurse)
3. Policy Creation	The culture at [our institution] is one of sort of scientific evaluation through the PUSA, plan, use, study, act, and that is that is assuming as we are talking that we are both kind of talking about change for betterment, so our process for change is through that process. There is [sic] elements of course of sort of this whole concept of policy development and knowledge translation that has to come from that but the front line clinicians seem to respond to and understand better that sort of simple tangible process of PUSA cycle. - Participants 5 (Administrator)
4. Implementation and Monitoring of Departmental Policies	Can we continuously audit all of the time to ensure that compliance? No, but do we pick and choose which ones we are going to audit more frequently? For sure. And it tends to be the ones that the staff are not so, are more resistant to the change. - Participant 3 (Administrator)
5. Actively Address Barriers Throughout the ED Policymaking Process	It's probably eight pages of policy and procedure. Same with our medical directives, it is probably … fifteen pages… and what happens is that people get totally turned off. … [I]t can take up to six months to turn a policy around which is very onerous and it kind of loses its sense of urgency sometimes, once you are waiting for sign-offs.... - Participant 13 (Nurse)

Step 1: Problem identification and motivation for change

Every policy and procedure, whether clinical or administrative, is developed to address a problem or improve patient care. In order to identify problems that require a policy solution, EDs in our LHIN used both quantitative and qualitative methods.

Most of our LHIN EDs mainly employed ad hoc methods, such as critical incident reviews and frontline staff discussions, to identify areas requiring change. Most participants emphasized the importance of recognizing departmental problems through a variety of avenues and being receptive to all employee concerns. Once problems are identified, solutions can be brainstormed and the need for a new policy creation can be systematically addressed. The underlying motivation for creating new policies, in any event, was to improve patient care and departmental safety. Quantitative methods that played into problem identification included ED departmental tracking tools (i.e., Distress Assessment and Response Tool, or DART). See Table 2 for an exemplar quote.

Although problem identification often provided the stimulus for policy creation, a number of participants explained that not all problems required a new policy solution. Depending on the identified problem, the solution may be better addressed by creating a more tangible bedside intervention such as a nursing protocol, or physician order set.

Step 2: Building a policy team

Once a problem was identified, the next step most participants described was to identify appropriate stakeholders that could drive and participate in the policy creation process. Based on the type of policy being created, a variety of internal and external stakeholders were identified. Although clinicians and administrators played a central role in policy creation, many study participants commented on the importance of engaging a variety of stakeholders and hospital committees. Most participants mentioned that their local departments had committees (e.g., Medical Advisory Council, or Quality Care Committee) that would participate heavily in the policy creation process. Some leaders highlighted the importance of creating smaller ‘working groups’ to focus on policy creation and the importance of having selective representation throughout this process. See Table 2 for an exemplar quote.

Ensuring appropriate stakeholder engagement throughout the process of policy creation was identified as being crucial to developing a practical policy. Most study participants advocated that policies should always be developed in conjunction with the stakeholders responsible for carrying out the final policy. Depending on the policy being developed, participants also identified the need for flexibility in refining the members of the working group, and adding and subtracting participants as needed.

Step 3: Policy construction

After the policy team is assembled, producing a new policy that is pragmatic and effective is the next step. As mentioned earlier, the literature on the process of departmental policy creation is scarce and most EDs in our LHIN did not have a common process for construction of a new policy.

Described Models for Policy Construction

**Figure 2:**
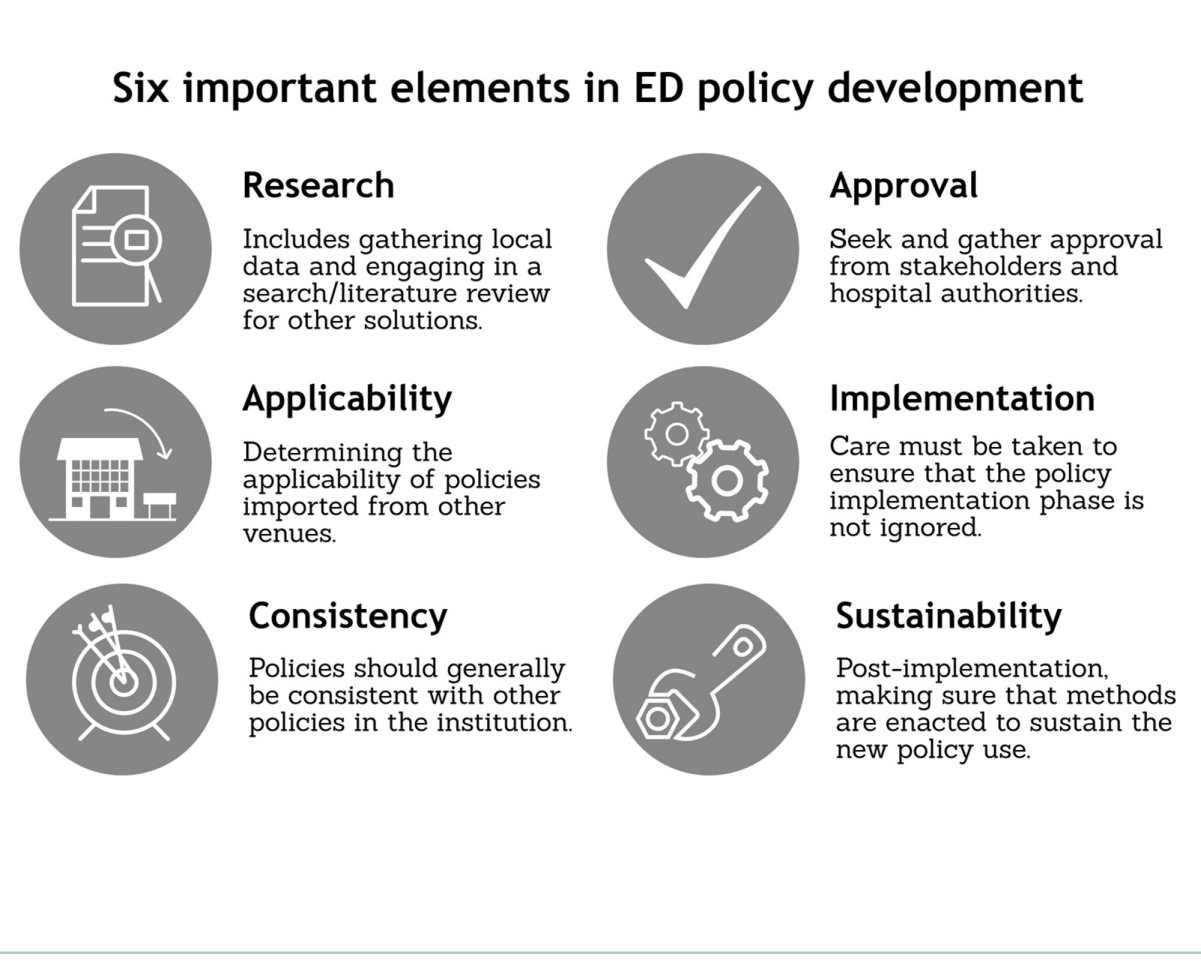
Six important elements in emergency department (ED) policy development. Six elements described by our participants which are important for ED policy development.

Our participants described a few different models for generating policy. Table 3 depicts a few policy construction models that were described by our participants. Figure 2 depicts the six most important elements as described by local policymakers.

**Table 3:** Policy creation models described by our participants.

Model Name	Description
Template-based	Participants mentioned that their institutions had policy departments that helped their clinical teams develop concrete policies that fit their hospital’s policy template.
Locally-derived processes	Through discussion, our study participants identified a variety of important steps for policy development including: research, approval, applicability, consistency, implementation, and sustainability. These steps are highlighted in Figure 1. There was significant variance in how explicitly these steps and concepts were described between sites, and how much each individual step was considered in the local policy environment.
PUSA or PDSA model	A number of respondents claimed that their policy team utilized a freeform style and that experience of team members helped guide the entire process. Systematic approaches were used less often, though the PUSA (Plan-Use-Study-Act) or PDSA (Plan-Do-Study-Act) model (6), was mentioned by five sites, though the degree to which this was a part of their policy creation was variable.

In order to ensure that clinical policies were in line with best practice, most interviewees identified the importance of research and literature review. From searching through guidelines and well-known academic resources (i.e., textbooks, websites), to conducting local focus groups and collaborating with nearby institutions, a variety of methods were utilized by policymakers to find solutions to their local problems. Smaller institutions relied heavily on larger centers with greater infrastructure to find policy solutions to their problems. Within the process of policy development itself, a number of participants stressed the importance of creating consensus amongst colleagues and ensuring that policies were practical for the front-line workers that would need to use them.

Although a number of study participants had heard of various policy frameworks, none of the interviewees were aware of or applied the conceptual frameworks from the literature. Opinions on whether a formal process of policy construction in the ED was required had mixed opinions with some policymakers suggesting that this could standardize the process, whereas others suggesting that policy construction is quite contextual and requires too much flexibility to follow any particular framework.

Step 4: Implementation and monitoring of departmental policies

Once new policies are developed and approved through the appropriate departmental channels, the task of dissemination to front-line staff begins. See Table 2 for exemplar quotes of how various leaders achieved this end.

Policy propagation was highly dependent on departmental size, infrastructure, and personnel preferences. The communication of policies occurs through a variety of channels including personalized emails, newsletters, departmental meetings, and in-person conversations. Larger hospitals utilized a combination of methods to reach their staff and often took months to roll out new policies. Smaller departments had the flexibility of utilizing more personalized approaches but are often limited by their human resources such as the number of educators they may have at their disposal.

In order to ensure that new policies are being appropriately implemented, compliance and ongoing follow-up are often essential. Almost universally, this was an area of weakness and difficulty in most departments due to the lack of resources and infrastructure. Although most institutions aim for complete compliance within a certain timeline after policy release, most policies’ uptake is not tracked in an objective manner.

Step 5: Actively address barriers throughout the policymaking process

Policymakers face many barriers during the policy creation and implementation. Our study showed that barriers can be generally categorized into knowledge-related, attitude-related, or internal and external barriers. See Table 2 for some exemplar quotes describing some of these barriers and how people tended to begin addressing some of these problems.

Barriers to change in the ED were highly dependent on the size, culture, and infrastructure of the department and organization. Ways to overcome barriers were often idiosyncratic to an organization and often involved some level of communication and progressive discipline. Four main themes emerged around barriers: a) Education and knowledge transfer; b) lack of a change culture; c) resource limitations; and d) cumbersome bureaucratic structures. Table 4 depicts these barriers and how our participants tried to combat them.

**Table 4:** Barriers to change and methods to combat them.

Barrier	Exemplar Quote	Description of Barrier	Methods Used to Combat Barrier
Poor capacity for education and knowledge transfer	“There [are] all of these excuses that are put forward when they don't want to work with something... You can have meetings, you can send emails, but people have to come or read them, right? … There are lots of docs who I have had problems with … [and] every time you talk to them it is like you have never said it to them before. And I mean what can you do. You don't really want to fire someone... you would like to see them improve.” - Participant 1 (Physician)	Difficulty in delivering education about policy change to staff	1. Personalized communication with employees that failed to adhere to departmental standards. 2. Establishment of regular academic processes, such as journal clubs and grand round lectures. Sites with these did not experience significant knowledge-related barriers. 3. Smaller institutions looked to larger counterparts to overcome knowledge barriers.
Lack of a change culture	“If you tailor a policy to the weakest link then it may not be the best, smartest policy… but at the same time if you make it ideal it will be too cumbersome... People will dig their heels in and again it goes back to that statement of culture eats process for breakfast... if the culture is such that no we are not going to do that or we are going to make it difficult... then it falls apart and we don't get anywhere.” - Participant 1 (Physician)	Departmental culture that promoted change and was flexible enough to adopt new solutions was key in instituting policy changes. Institutions without such a culture often met significant resistance to new policy implementation.	1. Identifying root of resistance to the policy. 2. Reflective review of the policy and evidence to support the reasoning behind the policy creation. 3. Strong leadership motivating change. 4. Informal discussions to gain individual stakeholder buy-in.
Resource limitations	“You have one educator for a huge stream, so she has all of mental health; she has all of the out-patient programs- and emerg. So to implement a change in practice, they are stretched thin” - Participant 7 (Administrator)	These limitations included capital resources, such as equipment and funding, and particularly in smaller institutions, also the lack of human resources and time to produce new policies.	1. Adapting policy from other nearby centres to local circumstances helped overcome personnel and time limitations.
Cumbersome bureaucratic structures	“There are levels of bureaucracy that we are just not aware of.. like we are going to come up with a form and you can't actually just come up with a form... we were told after the fact that we can't do that because you are asking [about] issues around employee rights... the unions got involved [and] I had to pull the survey. There are all of these levels of bureaucracy and we are starting to learn what it is that are actually the roadblocks” - Participant 8 (Physician)	Larger institutions especially had difficulties efficiently accommodating large numbers of stakeholders and committees while ensuring that all relevant parties were included. The onerous, length policy process was off-putting to many staff, whom further had many responsibilities and received no formal compensation for their policy roles.	Mixed teams of clinicians (with frontline knowledge) and administrators (with knowledge of the procedures for developing policies) contributed their strengths to different aspects of the policy process.

## Discussion

Qualitative research can allow us to better understand how knowledge may be translated to the bedside [10,11], yet few studies have been conducted with this end in mind [12]. Our results show that policy creation in the ED is a complex process that involves many moving parts and a variety of different stakeholders. In our particular study, we have described how policies are created in this busy and complex environment – and how while clinical evidence often guides the content of the policies, it typically has only a minor role in whether or not a policy is implemented well. Although EDs may be similar in terms of the pathologies and patients they see, departmental resources and policy protocols differ greatly even within one region within Ontario, Canada. The aim of our study was to identify similarities and differences and present common themes that policymakers could consider to make more evidence-based decisions in their line of work. We are hoping that by presenting the newly derived conceptual frameworks from our work we can clarify the processes that leaders might use when constructing new local policies within their units or departments.

Our proposed conceptual framework (Figure 1) highlights five crucial steps that may prove to be useful for both –those developing policies in their local departments, and may also inform scientists interested in assisting leaders to adapt new knowledge into their local policies by providing them with insights as to where new evidence is used by local policymakers. For example, understanding that problem identification is such a key component of the policy-generation process might suggest that a scientific team looking to create a toolkit for disseminating their latest findings to local policymakers might situate their new care bundle or package as a solution to a common problem experienced by local clinicians. Case-based examples that resonate with common bedside clinical problems may be more powerful to highlight and illustrate the need to incorporate new data into a local process/policy.

In Canada, there is currently no standardized process for ED departmental policy creation in our LHIN and, as such, there is considerable opportunity for policymakers to miss critical steps during this process. Although we understand that local context heavily influences the policymaking process, a standardized framework would ensure a more systematic approach for policymakers. In an era where clinicians stress the importance of evidence-based medicine, we strongly believe that policymakers should be held to the same standards as practicing clinicians. Similarly, knowledge producers such as scientists must also be aware of these key elements of policy development if they are to improve uptake of their work within the clinical environment [6,7].

Although many frameworks exist for the policy creation process, such as the Ottawa Model of Research Use or the Knowledge to Action framework, none of these were formally identified as being used in our local emergency departments. The most commonly mentioned approach was the PDSA (Plan-Do-Study-Act) model, however even this was discussed mostly conceptually rather than as a framework that was closely followed throughout the policy creation process. Our proposed conceptual framework (Figure 1) highlights five crucial aspects of policy formation that should be individually considered for policy formation in the ED regardless of the stakeholders or the type of process currently in use. These steps highlight the importance of not only the content of a given policy, but what must be considered for a policy to be adequately accepted and continued in practice. Some centers had already recognized some of these steps explicitly, while others mentioned aspects of these concepts without explicitly identifying them. Specific consideration of these steps should allow more uniformity in the approach to policy development and identification of potential difficulties of implementation while still allowing for local flexibility and recognition of barriers specific to individual hospital situations.

Our study also highlights the opportunities for collaboration when it comes to departmental policy creation. Academic tertiary care centers that have higher capital and resources are often more empowered when it comes to knowledge translation and policy development. They may also have access to in-house expertise that is unavailable in smaller centers. Smaller centers may benefit from collaborating with these institutions while altering policies as necessary to be applicable to their own local context.

Limitations

As a qualitatively designed study, our results are only as valuable as the credibility of the data provided by study participants. There were a total of 27 potential study participants identified through the ESSC but we were only able to recruit 15 of these to participate, despite multiple contact attempts. A second limitation was the exclusive use of physicians in our analytic team. Qualitative data such as our may have yielded alternative themes if it was interpreted by administrators or clinicians with alternate backgrounds, such as nurses. However, to ensure that our data was appropriately analyzed, we incorporated trainees (SS, TS) with little clinical leadership experience into our team in order to ensure that the other authors (TC, AP) remained reflexive.

Importantly, the authors would like to remind readers that qualitative studies are not meant to be statistically generalizable. Rather, this type of research seeks to provide new insights and understanding about the humans within our health systems and shine light on their thinking and behaviours [13]. By providing these insights, we hope our paper may contain certain insights about how healthcare policies are made at the local level, which we hope will be broadly applicable to a wide array of healthcare settings where policies are being actively developed [13].

## Conclusions

Departmental policy creation in emergency departments is a complex process that is contextually driven and responsive, but lacks allegiance to evidence-based medicine. If we are to strive for true evidence-based practice in our clinical environments, we must open the conversation about evidence-based policy development at both the bedside and in the boardroom. Our study identified common facilitators and barriers that policymakers face in their change processes. Due to the limited evidence on policy creation in the ED, our results suggest that there may be a greater need for policy-makers to develop a systematic approach for incorporating new clinical evidence or guidelines into local policies.
